# Identification and molecular characterization of *Subramaniula asteroides* causing human fungal keratitis: a case report

**DOI:** 10.1186/s12879-021-05768-7

**Published:** 2021-01-18

**Authors:** Rosario Cultrera, Riccardo Torelli, Caterina Sarnicola, Daniela Segala, Andrea Mengoli, Giuseppina Chiaretto, Paolo Perri, Maurizio Sanguinetti

**Affiliations:** 1 Department of Morphology, Surgery and Experimental Medicine, Infectious Diseases Unit, University ‘S. Anna’ Hospital of Ferrara, Via Aldo Moro 8, 44124 Ferrara, Italy; 2 Dipartimento di Scienze di Laboratorio e Infettivologiche, Fondazione Policlinico Universitario “A. Gemelli” IRCCS, Largo Agostino Gemelli 8, 00168 Rome, Italy; 3 Department of Biomedical and Surgical Sciences, Ophthalmology Unit, ‘S. Anna’ University Hospital of Ferrara, Via Aldo Moro 8, 44124 Ferrara, Italy; 4 Clinical Microbiology, ‘S. Anna’ University Hospital of Ferrara, Via Aldo Moro 8, 44124 Ferrara, Italy; 5 Dipartimento di Scienze Biotecnologiche di base, Cliniche Intensivologiche e Perioperatorie, Università Cattolica del Sacro Cuore, Largo Agostino Gemelli 8, 00168 Rome, Italy

**Keywords:** Fungal keratitis, *Subramaniula asteroides*, Molecular identification, β-Tubulin gene, Isavuconazole, Case report

## Abstract

**Background:**

Keratitis due to by filamentous fungi are not easy to diagnose thus causing a delay in correct therapy. There are many descriptions of keratitis due to *Candida, Fusarium* and *Aspergillus* genera. *Subramaniula* genus has only recently been reported to cause human infections and there are few descriptions of eye infections due to this filamentous fungus. Diagnosis of fungal keratitis is usually based on microscopic and cultural techniques of samples obtained by corneal swabbing or scraping. Considering the amount of time required to obtain culture results it is wise to use other diagnostic methods**,** such as molecular analyses. Therapeutic options against these fungi are limited by low tissue penetration in the eye due to ocular barriers. We describe the first case of *S. asteroides* human keratitis treated with isavuconazole.

**Case presentation:**

We describe a rare case of fungal keratitis unresponsive to antimicrobial treatment in a 65-year-old male patient without a history of diabetes or immunological diseases. He reported that the onset of symptoms occurred during a long holiday in Cape Verde Island. Initial treatment with topical antibiotics associated to steroids were ineffective, allowing a slow clinical progression of disease to corneal perforation. On admission in our Hospital, slit-lamp examination of the left eye showed conjunctival congestion and hyperemia, a large inferior corneal ulceration with brown pigment, corneal edema, about 3 mm of hypopyon and irido-lenticular synechiae. The slow clinical progression of the disease to corneal perforation and the aspect of the ulcer were consistent with a mycotic etiology. Molecular methods used on fungal colonies isolated by Sabouraud’s dextrose agar cultures allowed the identification of *Subramaniula asteroids* from corneal scraping. Antimicrobial test showed a good susceptibility of this filamentous fungus to voriconazole and isavuconazole. Moreover, this fungal keratitis was successfully treated with isavuconazole, without side effects, observing a progressive clinical improvement.

**Conclusions:**

Molecular methods may be useful for the identification of filamentous fungal keratitis on scraping samples thus shortening the time of diagnosis. Systemic therapy by isavuconazole could be useful to treat the filamentous fungal keratitis, reducing the possible adverse effects due to the use of voriconazole by systemic administration.

## Background

Fungal keratitis (FK) is considered responsible for 30–60% of infectious keratitis in humans with serious damage to vision that often require corneal restroom surgeries**,** and sometimes, corneal transplantation**,** enucleation or evisceration [1–3].. They have become more frequent over the last four decades favoured by conditions such as diabetes, chronic ocular diseases, immuno**-**compromised patients, increased use of contact lens, topical steroids and antibacterial drugs, as well as an increase in surgical procedures [1, 2, 4–6].

FKs can be mainly caused by *Candida, Fusarium* and *Aspergillus* species [1, 3, 7]. Usually, FK is due to fungal access into the corneal stroma through a defect in the epithelium and trauma represents a frequent event. Diagnosis of fungal keratitis is commonly based on standard methods such as microscopic and cultural techniques of samples obtained by corneal swabbing. Identification of filamentous moulds is based mostly on microscopic examination of sporulating colonies. These methods could result inadequate in confirming a fungal agent**,** because fungi penetrate deeper layers of the cornea. Moreover, corneal culture is scarcely sensitive and requires long growth times, usually taking 1 to 35 days, especially when an antibiotic resistance test should be performed. In vivo confocal microscopy may be a helpful clinical adjunct but is still not available everywhere and lacks sufficient resolution to identify fungal hyphae [8, 9].

Molecular methods have been developed, demonstrating their usefulness as a rapid, highly sensitive and accurate diagnostic tool [10], though the latter are not easy and limited to only a few laboratories that are able to use these techniques. To avoid negative results due to deep infiltrates, corneal scrapings are recommended, and these samples should be analysed by traditional and molecular techniques. Direct-PCR on biological sample is highly sensitive and could be considered a good method for FK diagnosis as it reduces the time needed, taking 2 to 3 h. The obstacle to using PCR is that it requires very specialized equipment that may not always be available. Therefore, Kuo et al. [11] suggested implementing a dot hybridizaton assay, which is highly sensitive and can detect a wide variety of fungi. This assay used PCR to first amplify the highly conserved fungal 5.8S rRNA gene before adding it to immobilized oligonucleotide probes specific for fungi fixed to a nylon membrane. Detection by this dot assay, which could be seen with the naked eye, was reported to have been 100% sensitive and 96.7% specific for fungi identification.

FKs treatment is difficult because the diagnosis is often delayed and no antifungal drugs for topical eye use are commercially available; most used topical galenic antifungals often fail to achieve an adequate control of keratitis. Once fungi reach the deep stroma, they can penetrate into the anterior chamber and there is a high risk of endophthalmitis that can lead to ocular enucleation. One study suggested early deep anterior lamellar keratoplasty as a possible safe therapeutic approach to effectively eradicate fungal keratitis affecting the optic zone and poorly responsive to medical treatment, thus avoiding corneal transplant [12]. Therapeutic options against these fungi are limited by low tissue penetration in the eye due to ocular barriers. Most of the inside of the eye lacks blood vessels and the outer and inner blood-retinal barrier**,** which limits the influx of drugs into the retina and vitreous regions, requires the systemic administration of high doses to achieve therapeutic concentrations within the eye. Moreover, blinking and tear film turnover limit**s** the residence time of a drug and the access to the deeper structures of the eye is hindered by corneal epithelium and stroma with varying lipophilicity.

We report a rare case of keratitis due to *Subramaniula asteroides* [13] in a patient without co-morbidities and with no reported eye trauma, identified by molecular methods and successfully treated with systemic isavuconazole associated to topic voriconazole.

## Case presentation

A 65-year-old male patient without **a** history of diabetes or immunological diseases presented to the Emergency Unit of University Hospital S. Anna of Ferrara, Italy, with photophobia and ocular pain in the left eye. The patient had just returned from a long holiday in Cape Verde and he reported that the onset of symptoms had occurred about 20 days earlier with the sensation of a foreign body in the eye, without any previous ocular trauma. In a Medical Centre of Cape Verde Island, he was diagnosed with a keratitis in the left eye, initially treated with tobramycin 0.3% and dexamethasone 0.1% eye ointment for a week, then discontinued because of the worsening of his condition. Then oral amoxicillin 875 mg and clavulanic acid 125 mg tid and topical ofloxacin 0.3% eye drops qid were administered for the following 10 days. Since the symptoms were more severe and the visual acuity reduced, the patient decided to return to Italy.

**Fig. 1:**
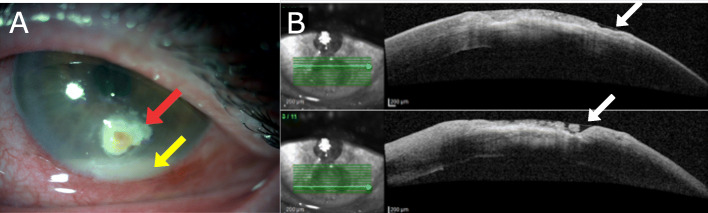
**a** Slit lamp picture taken on initial presentation in our Hospital showing an inferior corneal ulceration with brown pigment (red arrow) and about 3 mm of hypopyon (yellow arrow) in the left eye. **b** Anterior segment optical coherence tomography (AS-OCT) showing the depth of the ulcer (white arrow)

On admission to our Hospital the best correct visual acuity was 20/200 at distance in the left eye. Slit-lamp examination of the left eye showed conjunctival congestion and hyperemia, a large inferior corneal ulceration with brown pigment, corneal edema, about 3 mm of hypopyon and irido-lenticular synechiae (Fig. 1). The posterior segment was not visible, ultrasonography did not show any sign of intraocular infection. Corneal scrapings were obtained for microbiological analyses. Sample obtained by scraping was directly inoculated onto chocolate agar plate (Vacutest® KIMA) and sent to the Clinical Microbiology Laboratory of University Hospital of Ferrara and incubated at 37 °C with 5% of CO_2_ atmosphere.

Since the clinical picture was suggestive of a fungal keratitis, pending the outcome of microbiological tests, the patient immediately started treatment with fortified topical tobramycin 14 mg/ml qid, moxifloxacin 0.5% eye drops qid, cyclopentolate 1% tid and voriconazole 1% every 2 hours [14]. In addition, oral voriconazole was administered at the loading dose of 400 mg bid on days 1 and 2 and subsequently at the dose of 200 mg bid from day 3. Blood cell count and liver and kidney function blood tests were monitored every 15 days.

**Fig. 2:**
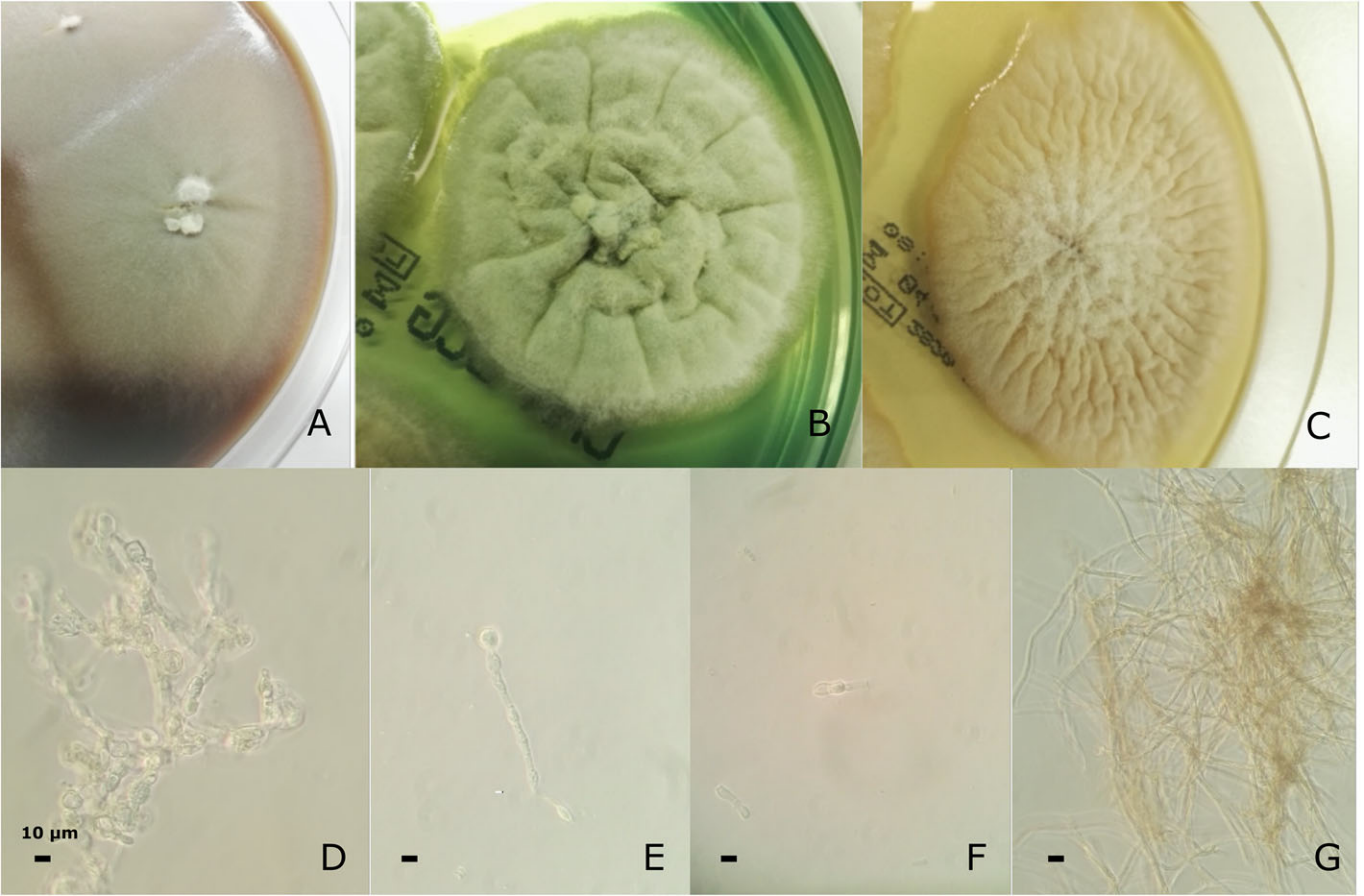
Macroscopic view colonies after one week of incubation on: **a** Chocolate Agar plate (CHOC); **b** BCG agar plate; **c** Sabouraud Dextrose Agar plate; **d**-**g** Microscopic view 0,25% Lugol stain. Microscopic description: hyphae broad, septate, hyaline, turning dark brown with age. Conidiophores phialidic, terminal or intercalary, short, hyaline, obclavate or cylindrical. Conidia hyaline, unicellular, obovoidal or ellipsoidal

Forty-eight hours after corneal scraping fungi colonies were observed without any bacterial growth. Microscopic examination evidenced hyaline filamentous forms with clear mycelium. Growth was shown at 30 °C and 37 °C after 1 week of incubation on Chocolate Agar plate (CHOC), BCG agar plate, and Sabouraud Dextrose Agar plate (Fig. 2a,b,c). Microscopic observation showed hyphae broad, septate, hyaline, turning dark brown with age. Conidiophores phialidic, terminal or intercalary, short, hyaline, obclavate or cylindrical. Conidia hyaline, unicellular, obovoidal or ellipsoidal (Fig. 2d, e, f, g).

Cultured samples on Sabouraud’s agar plates were sent to the Laboratory of Mycology at Policlinico “A. Gemelli” of the Catholic University of Rome for biomolecular analyses to identify the filamentous fungus obtained by cultures [15, 16].

Molecular methods allowed the identification of *Subramaniula asteroides* by fungal DNA extraction (using Plant extraction kit, Qiagen®), PCR amplification of β-tubulin gene (*βtub*) by specific primers and Sanger sequencing method of fungal gene [13, 17, 18]. PCR products were sequenced and compared in GenBank database (https://blast.ncbi.nlm.nih.gov/Blast.cgi). The *βtub* sequence shared 99% identity with the reference sequence (KP900696.1) for *Subramaniula asteroides* (Strain CBS 128679).

Antifungal susceptibility testing on the case strain was performed by broth micro**-**dilution according to CLSI methods for filamentous fungi (Sensititre™ YeastOne ITAMYUCC, Thermo Scientific) [10]. This test showed a low minimal inhibitory concentration (MIC) of *S. asteroides* for isavuconazole and the other triazoles, echinocandins and amphotericin B (Table 1).

**Table 1:** Susceptibility of *Subramaniula asteroides* to antifungal drugs tested

Antifungal drugs	***MIC***
Amphotericin B	0.25
Anidulafungin	0.125
Caspofungin	0.5
Micafungin	0.125
Voriconazole	0.06
Posaconazole	0.06
Itraconazole	0.06
Isavuconazole	0.06

Antifungal susceptibility testing on the case strain was performed in triplicate by broth microdilution according to CLSI methods for filamentous fungi (Sensititre™ YeastOne ITAMYUCC, a modified panel YO10 with isavuconazole instead of 5-fluorocytosine, Thermo Scientific). Sensititre YeastOne was tested for efficacy in *Aspergillus* spp. and non-*Aspergillus* molds MIC valuation as previously described [19–21]. MIC values of the triazoles for *Candida krusei* ATCC 6258, *A. fumigatus* ATCC MYA-3626, and *A. flavus* ATCC 204304, which were used as quality control isolates, were all within the expected ranges (data not shown). The MICs of the antifungal drugs amphotericin B, anidulafungin, caspofungin, micafungin, voriconazole, posaconazole, itraconazole, isavuconazole were 0.25, 0.125, 0.5, 0.125, 0.06, 0.06, 0.06, 0.06 μg/mL, respectively (Table 1). Unfortunately, there are no clinical breakpoints available for *Subramaniula asteroides*. However, CLSI epidemiological cutoff values for antifungal susceptibility testing for *Aspergillus* spp*.* have recently been released [22]. Regarding these molds, the document shows ECV (μg/mL) of amphotericin B, caspofungin, isavuconazole, itraconazole, posaconazole, voriconazole.

Due to the scarce improvement of the keratitis and considering the inability to determine the ocular tissue concentration levels of the drugs in the patient, voriconazole was stopped and isavuconazole was started at the loading oral dose of 200 mg tid for the first 48 h**,** continuing with 200 mg per day. Ten day**s** later, hypopyon had been reduced and symptoms were improved. Over the next 4 weeks the cornea had epithelized, and the inflammation was solved. Oral isavuconazole, topical antimicrobials and cyclopentolate were discontinued after 6 weeks.

**Fig. 3:**
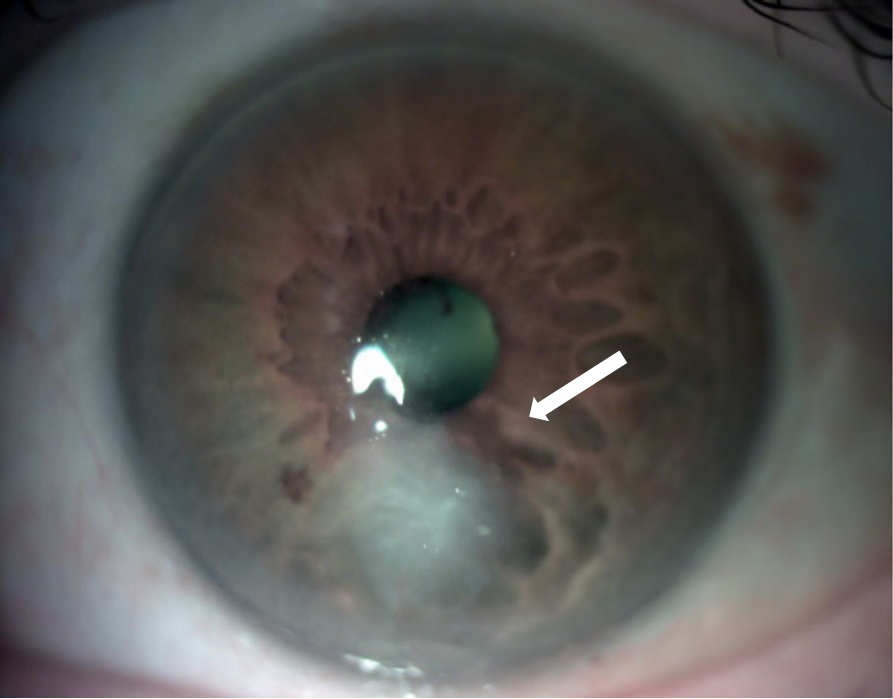
Two months after treatment non signs of active infection. A residual para-central corneal scar (white arrow) limits the visual acuity to 20/50

The fungal corneal ulcer due to *S. asteroides* was successfully treated**;** the best correct visual acuity 2 months after treatment was 20/50 with a residual corneal scar (Fig. 3). Periodic eye tests showed a progressive improvement.

## Discussion and conclusion

Filamentous fungi may be implicated in fungal keratitis in humans**,** which is particularly widespread in tropical countries. It is very important that a specific diagnosis be made as quickly as possible to ensure a prompt institution of adequate antifungal therapy.

*Aspergillus* spp. and *Fusarium* spp. are the most common species responsible for human keratitis. We report a first case of keratitis due to *S. asteroides* treated with isavuconazole. *S. asteroides* is a filamentous fungus present in the natural environment and strongly associated with traumatic eye injury or skin infections [13, 18]. *Subramaniula asteroides* belongs to the phylum Ascomycota, family Chaetomiaceae and genus Subramaniula. The members of this family are ascosporulating fungi. The Chaetomiaceae (*Subramaniula asteroids*, *Subramaniula obscura* and mainly *Chaetomium anamorphosum*) were considered opportunistic pathogens rarely causing skin infections and keratitis due to their ability to grow at high temperatures [18]. Apart from these reports, the role of this fungus as a human pathogen is largely ignored.

In our case, the slow clinical progression of disease to corneal perforation and the aspect of the ulcer were consistent with a mycotic etiology. The patient was initially treated with a topical antibiotic associated to a steroid. Because of the poor response, further treatments were prescribed with prolonged cycles of topical antibiotics and steroids without any benefits and consequent loss of the visual acuity. Corneal scraping performed at the Ophthalmologic Clinic allowed the identification of filamentous moulds by preliminary microscopic examination. There are findings that the delay of diagnosis could require surgical treatment up to the point of an eventual ocular evisceration, as described in *Fusarium* spp. keratitis [7].

Only molecular techniques allowed the exact identification of *S. asteroides* as responsible for keratitis. Previous reports of *S. asteroids* human infections are described in a case of endophthalmitis in a patient with non-insulin dependent diabetes mellitus after an eye trauma treated with topical amphotericin B deoxycholate in combination with fluconazole for 42 days [14], in a male patient with sinusitis without other disorders [14], and in a case of keratitis by *S. asteroides* after a corneal trauma [23].

This is to our knowledge the first case of *S. asteroides* human keratitis treated with isavuconazole. This drug was able to eradicate the infection of *S. asteroides* keratitis after a poor response to a first course of voriconazole. Prolonged topical voriconazole treatment in fungal keratitis can induce ocular surface dysplasia [24]. Moreover, patients who received voriconazole had a corneal perforation or required therapeutic penetrating keratoplasty [25]. Isavuconazole was well tolerated, confirming the data of less hepatobiliary, eye and skin disorders. The favourable outcome allows us to hypothesize that the ocular concentration of isavuconazole is sufficient to eradicate the fungal infection, as suggested by Schmitt-Hoffmann A. in a study about the tissue concentrations of isavuconazole in the eye and in the lacrimal glands of rats [26]. Unlike the other azoles, to our knowledge there are no data regarding the concentration of isavuconazole in the human eye.

## Data Availability

The datasets supporting the conclusions of this article are included within the article and additional files. The datasets used during the current study are available from the corresponding author on reasonable request.

## Abbreviations

tid: Ter in die
qid: Quarter in die
*S. asteroides*: *Subramaniula asteroides*
MIC: Minimal inhibitory concentration
*Btub*: β-tubulin gene
CLSI: Clnical and Laboratory Standards Institute, 950 West Valley Road, Suite 2500, Wayne, PA 19087, USA
